# Infant and child mortality in the Netherlands 1935–47 and changes related to the Dutch famine of 1944–45: A population-based analysis

**DOI:** 10.1080/00324728.2023.2243913

**Published:** 2023-09-12

**Authors:** Ingrid J. J. de Zwarte, Peter Ekamper, L. H. Lumey

**Affiliations:** 1 Wageningen University & Research; 2 Netherlands Interdisciplinary Demographic Institute; 3 University of Groningen; 4 Columbia University; 5 Netherlands Institute for Advanced Study

**Keywords:** Dutch Hunger Winter, famine, food distribution, German occupation, infant and child mortality, stillbirths, the Netherlands, urban advantage, World War II

## Abstract

Precise estimates of the impact of famine on infant and child mortality are rare due to lack of representative data. Using vital statistics reports on the Netherlands for 1935–47, we examine the impact of the Dutch famine (November 1944 to May 1945) on age-specific mortality risk and cause of death in four age groups (stillbirths, <1 year, 1–4, 5–14) in the three largest famine-affected cities and the remainder of the country. Mortality during the famine is compared with the pre-war period January 1935 to April 1940, the war period May 1940 to October 1944, and the post-war period June 1945 to December 1947. The famine’s impact was most visible in infants because of the combined effects of a high absolute death rate and a threefold increase in proportional mortality, mostly from gastrointestinal conditions. These factors make infant mortality the most sensitive indicator of famine severity in this setting and a candidate marker for comparative use in future studies.

## Introduction

Excess mortality is an important indicator of a famine’s severity. Analysing historical mortality data is not only important for assessing the overall impact of famine but also for developing measures for famine relief and prevention. Yet for the vast majority of historical famines, scholars have emphasized that estimating excess deaths is a difficult undertaking, because representative statistical data are often missing, incomplete, or otherwise unreliable due to ambiguous registration practices or statistical manipulation (De Waal 1989; Ellman 2000; Ó Gráda 2010; Wheatcroft and Ó Gráda 2017). Even if death records from famine-exposed localities are available, demographic conclusions are frequently compromised by the paucity of data for baseline reference periods, the unknown age composition of the population, or the difficulty of taking into account the effects of migration (Dyson 1991; Cherepenina 2005; Hionidou 2006).

The relative vulnerability of infants and children to famine remains a much-discussed point in the literature in view of the likely short- and long-term health effects of famine exposure at young ages (Lumey and Vaiserman 2013). In most famines of the nineteenth and twentieth centuries—and likely earlier famines as well—the main victims *in absolute numbers* have been young children aged 0–4 years and adults beyond middle age. These prevalent mortality patterns can be ascribed to biological mechanisms. Generally speaking, young children and older people are more susceptible to infections and injuries, and they recover more slowly from disease; famine exposure enhances these normal patterns (Lumey and Van Poppel 1994). However, the greatest *proportional* mortality increase has often been among older children, as mortality in normal times is lowest at these ages and small absolute increases can have large relative effects (Ó Gráda 1999, 2010). For example, during famines in Ireland in 1846–50, Finland in 1866–68, the Union of Soviet Socialist Republics (USSR) in 1922, Ukraine in 1932–33, Bengal (India) in 1943–44, Greece in 1941–44, and Darfur (Sudan) in 1984–85, the largest proportional mortality increases were reported in later childhood, at ages 5–14 (De Waal 1989; Dyson 1991; Pitkänen and Mielke 1993; Ó Gráda 1999; Adamets 2002; Vallin et al. 2002; Hionidou 2006).

Some twentieth-century famines have shown a different age-specific mortality pattern. During the 1947 USSR famine, proportional mortality in males showed the highest increase among infants and older people and the lowest increase around age 10 (Adamets 2002). Similarly, Hionidou’s (2006) account of age- and sex-specific annualized famine deaths in Greek localities during the occupation years, 1941–44, showed no proportional mortality increase among children aged 10–14 in towns on the island of Chios. She ascribed these ‘exceptional’ local death rates to well-functioning soup kitchens providing relief to schoolchildren during the famine. A limitation of the findings from the USSR and Chios is that they are based on aggregated, annualized deaths and on changes in the proportion of famine deaths in different age groups, whereas the age structure for the population at risk remains unknown.

There is a suggestion that the age-specific mortality pattern during the Dutch ‘Hunger Winter’ famine of 1944–45 may have been similar to that reported for the USSR and Chios. The Dutch famine was caused by the disruption of food supplies to the large cities in the western Netherlands during the final months of German occupation in the Second World War. Ekamper et al. (2017) demonstrated that during the famine, the lowest mortality in absolute numbers was seen at ages 1–14 and that this age group also showed the lowest increase in proportional mortality. The precise impact of the Dutch famine on children of various ages remains unclear, however, as Ekamper et al. did not report under-five (child) mortality as a separate subgroup. Further analyses by De Zwarte (2020) suggest that the lowest proportional mortality increase during the Hunger Winter was seen among older children, but her data were limited to the years 1944–46 and did not include age-specific causes of death for all localities.

To overcome the limitations of previous studies, we assembled nationwide data for the Netherlands on monthly stillbirth rates and monthly age-specific mortality for infants (aged <1 year) and children at ages 1–4 and 5–14 in the period 1935–47. Data were extracted from a variety of published sources. Our observations covered the Dutch Hunger Winter of 1944–45, and we added selected pre-famine and post-famine years for the comparison of mortality patterns. The famine was concentrated in the urban areas of the German-occupied western Netherlands and was well defined in time and place (see Ekamper et al. 2020). We examined mortality in the three main famine cities in the western Netherlands separately from mortality in the remainder of the Netherlands. Several characteristics of the assembled data are worth emphasizing. Mortality registration nationwide was already highly standardized before the 1940s and continued largely uninterrupted during the Hunger Winter. This has not often been the case during famines else-where. Furthermore, the data on births and age-specific mortality were specific enough to be analysed at the local level. We estimated the population age structure over the observation period from national census data and vital statistics reports on births and age-specific deaths in intermittent years. Outward migration during the famine was negligible.

The setting of the Netherlands provides a unique opportunity to examine in more detail than before the absolute and relative vulnerability of infants and children to famine. It allows for the estimation of famine-related excess deaths and changes in proportional mortality among specific age groups, using well-defined populations of infants and children with and without famine exposure. With this information we aim to identify a robust marker of famine severity in acute settings that could be used in future comparative studies.

### Historical setting of the Dutch famine

#### Pre-war mortality

Mortality rates in the Netherlands before the Second World War were among the lowest in the world (Hart 1993). In 1939, overall mortality was 8.7 per 1,000 population, life expectancy was 65.7 years, and infant mortality (deaths <1 year) was 33.7 per 1,000 live births (Banning 1946; Hemmes 1947). In the years before the war, infant mortality in the Netherlands was even lower than in Sweden, Norway, or Denmark (De Haas 1956; Chase 1967).

#### Famine-related mortality

Initial estimates of excess mortality during the Dutch famine were based on a comparison of the absolute number of deaths in the first half of 1945 with deaths in the first half of 1944 in 12 famine-exposed municipalities (Centraal Bureau voor de Statistiek (CBS) 1945; see also Dols and van Arcken 1946). Subsequent mortality estimates varied greatly depending on the regions and time windows included (Jong 1981; Trienekens 1985; Barnouw 1999), on the reported data sources (Futselaar 2008), and on the specific interpretation of the nature of deaths officially reported by doctors as being ‘from hunger’ (Burger et al. 1948; Stein et al. 1975). These studies reported numbers of famine-related deaths to have been anywhere between 10,000 and ‘tens of thousands’.

Recent estimates of wartime-related mortality in the Netherlands have been presented by Ekamper et al. (2017), who used nationwide death registry data that included date and cause of death, taking seasonal patterns and the population age composition into account. Based on these data, Ekamper et al. (2017) suggested an excess of 23,000 deaths in the six largest cities in the famine-affected west of the country between September 1944 and July 1945, most of which likely resulted from undernutrition and related causes of death. They also suggested 12,500 excess deaths in the rural west and 26,500 excess deaths in the remainder of the country not affected by the famine. These non-famine deaths most likely resulted from military activities related to the liberation of the country in 1944–45.

With regard to stillbirths, previous studies have shown that perinatal losses in the famine-struck western Netherlands paradoxically remained below those in non-famine areas (Stein et al. 1975). Hart (1993, p. 46) ascribed these comparatively favourable stillbirth rates in the famine region to advantageous socio-economic factors: the ‘successive intergenerational improvements in maternal health status’ in the urbanized western Netherlands. The longer-term patterns necessary to understand these stillbirth rates have, however, not yet been investigated, as Stein et al.’s (1975) and Hart’s (1993) data included only the years 1944–46.

#### Effects of in utero famine exposure

Regarding in utero exposure to famine and its immediate consequences, some significant findings from the Netherlands are worthy of attention. The seminal work by Stein et al. (1975) showed that perinatal mortality was higher among those with famine exposure in early gestation and that mortality in the first three months of life increased among those with famine exposure in late gestation, as the latter also affected birthweight and compromised child resilience. Furthermore, mortality 90–365 days post-partum was increased by the combined effects of prenatal famine exposure in late gestation and postnatal exposure to summer epidemics. Longer-term impacts of prenatal famine exposure in the Netherlands on overweight, diabetes, schizophrenia, and mortality at adult age have been reported extensively elsewhere (e.g. Lumey et al. 2011, 2021; Ekamper et al. 2014).

#### Pre-war nutrition

In the interwar period, the Netherlands enjoyed a well-functioning food system and food security (Trienekens 1985). Nutrition estimates among accurately defined populations are hard to find, but a house-hold budget survey among 598 families in the Netherlands in 1936 suggested that the average calorific intake among manual labourers was about 3,260 kcal per day, compared with an average consumption of 3,420 kcal/day for agricultural labourers and 3,965 kcal/day for farmers (CBS 1938; Van der Bie 2001).

#### Food rationing during the war

Contrary to the situation in most other European countries occupied by Nazi Germany, the invasion and occupation of the Netherlands in May 1940 did not immediately lead to problems with the food supply. Hitler appointed a German civil administration in the occupied Netherlands and instructed staff to maintain and adjust pre-war economic structures as needed to merge Dutch industry with the German war economy (Blom 1997). Dutch senior bureaucrats remained in office to ensure that the agricultural transition to self-sufficiency as planned before the war was achieved in an orderly manner. Working closely with the agricultural sector, the Dutch food administration developed an advanced rationing system, introducing this as early as 1939 in anticipation of possible food shortages in the event of war. During the war, the Dutch food administration limited black-market trade and negotiated relatively low export demands with Nazi Germany (Trienekens 1985, 2000; Klemann 2002). These measures prevented serious shortages of food in the Netherlands until 1944, with official civilian rations only slighter lower than those in Germany but much higher than in those in neighbouring Belgium or France (Lindberg 1946).

After the German invasion, more and more food items were included in the rationing system. By April 1941, nearly all foodstuffs had been included; with the exception of fruit, vegetables, and fish, none could be legally purchased without ration coupons (De Jong 1976). The Dutch food administration set the rations at a level assuming that house-holds would be able to add an additional 20 per cent of calories from informal and extralegal channels, including black-market trade. From the summer of 1940 onwards, soup kitchens became available for those who were not able to prepare meals at home, although they remained unpopular until the onset of the Hunger Winter in 1944 (Dols and van Arcken 1946).

Because of the well-functioning rationing system, the calorific value of the weekly food rations did not deteriorate much between 1941 and 1943. Food rations were allocated at the individual level and depended on the age and profession of each person. The system did not differentiate based on sex. Following changes in war-related circumstances, the official rations of all age groups began to decline in early 1944, although children’s rations were relatively less affected than those of adults (De Zwarte 2020).

There are different views on the nutritional value of the Dutch wartime diet. From his extensive studies of the wartime rationing system, Trienekens (1985, 2000) concluded that the Dutch diet was not only quantitatively and qualitatively sufficient until the start of the famine but may even have been ‘healthier’ than the pre-war diet because of its lower fat content and the increased consumption of vegetables and fibre. By contrast, Futselaar (2008) has argued that the typical diet already had serious nutritional shortcomings before the Hunger Winter and that this caused a significant increase in infectious diseases and mortality, especially among children. Annual measurements of about 7,000 schoolchildren in Amsterdam indicated that weight losses in this group started in 1941 and that in 1944, 13-year-old boys and girls were on average 2 and 5 cm shorter, respectively, and 3 kg lighter than before the war (Tuntler 1945). This suggests possible nutritional deficiencies before the famine.

#### Food rationing and additional food sources during the famine

The famine was well defined in place and time. It was concentrated in the urbanized western Netherlands, started in November 1944, and ended in May 1945 with the surrender of the German forces to the Allies. The famine was most severe in the three largest western cities—Amsterdam, Rotterdam, and The Hague (Ekamper et al. 2020)—with a combined population of around 1.8 million.

The well-defined boundary of the famine area was the cumulative effect of several transportation and distribution difficulties following the Allied liberation of the south of the Netherlands. In September 1944, the Allied advance stagnated at the River Rhine following the loss of the Battle of Arnhem. The northern part of the Netherlands remained occupied by the German forces until the spring of 1945, separating the western Netherlands from three major food-producing provinces as well as from the Dutch mining area in the south that normally provided coals for power and heating. The further impacts of a national railway strike, a temporary German embargo on inland shipping, a heavy frost period, severe fuel shortages, and the German requisitioning of transportation means resulted in increased food shortages and ultimately led to famine in the three western provinces (North Holland, South Holland, and Utrecht), inhabited by 4.3 million people, of whom 2.6 million lived in non-rural conglomerations (De Zwarte 2020).

**Figure 1:**
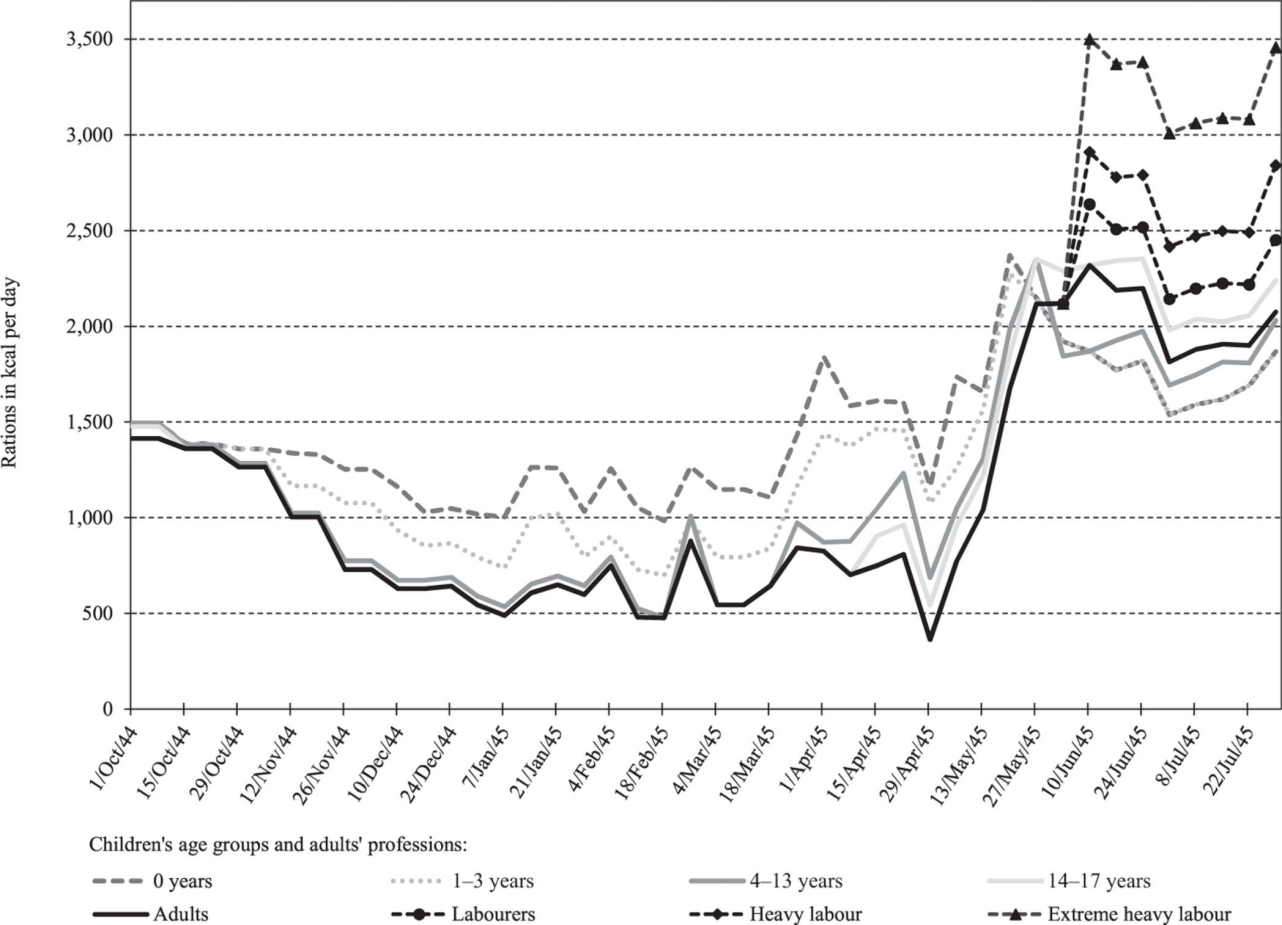
Weekly rations in kcal/day in the western Netherlands for different age groups and professions, October 1944 to July 1945 *Source*: National Archives of the Netherlands, Collection 2.11.23.02, inv.no. 192. See also: De Zwarte (2020).

By the end of November 1944, official rations in the occupied western Netherlands—which remained in the hands of Dutch senior officials—had dropped below 750 kcal/day for all people aged four or over (Figure 1). From that point onwards, the German civil authorities actively cooperated with the Dutch food administration to avoid further escalation of the famine conditions (De Zwarte 2020). Nevertheless, a period of winter frost caused a first low point of 500 kcal/day in January 1945, due to the loss of inland shipping possibilities. The lowest point was reached in late April and early May 1945, after the western Netherlands had become isolated from the rest of the country following the Allied liberation of the north-eastern provinces. From April 1945 onwards, the famine-affected western provinces were completely cut off from all major food-producing provinces.

Figure 1 shows that from October 1944 to June 1945, infants and toddlers were entitled to higher rations than adults. Children aged 4–17 were entitled to virtually the same rations as adults. Rations for children aged 4–13 increased somewhat for several weeks in the first months of 1945, thanks to limited Swedish and Swiss Red Cross relief allocations in the occupied areas. These Red Cross supplies were also used to provide young and expectant mothers with extra rations of oatmeal and milk powder. Although pregnant women were entitled to 1,114 kcal/day, Allied military surveys indicated that in February 1945 they received only 731 kcal/day (De Zwarte 2020). Differentiation between adult rations based on profession (i.e. labour intensity) returned only after June 1945.

Food distribution during the famine was also organized by local non-governmental relief organizations. These local relief actions offered two types of relief with a direct impact on children’s nutritional status. First, during the famine an estimated 33–50 per cent of urban school-aged children received three or more extra meals each week from school soup kitchens (De Zwarte 2018). Second, in early 1945 over 40,000 children aged 4–15 (about 9 per cent of the population at risk) were evacuated from the famine-affected cities to the north-eastern part of the country, where they stayed in foster families until after liberation (De Zwarte 2016). Both types of relief intervention prioritized physically malnourished children in the famine cities, based on their weight, height, sex, and age. By the end of February 1945, most relief efforts in the cities began to include young children (aged 1–3) as well.

## Materials and methods

### Data sources

During the war, and in particular during the last stages of the war, population registration and death registration (including causes of death) in the Netherlands were much less complete, accurate, and reliable than in normal times. Initially, the statistical activities of the Netherlands Central Bureau of Statistics (CBS) during the war continued as normal up to 1943 (van Maarseveen 1999), but in 1944 and 1945 data quality suffered. Especially from the second half of 1944, when the battle for the liberation of the country started, statistical registration was affected by under-registration of deaths and many more cases being registered with unknown cause of death than previously. After the war, CBS started to reconstruct the population statistics, supported by the 1947 Population Census (CBS 1955; Van Maarseveen 1999).

For this study, we used as our main data source the monthly CBS statistical bulletins (CBS 1935–47) that show the number of deaths in selected age groups (stillbirths, <1 year, 1–4 years, and 5–14 years) for the entire country, at the regional level (provinces), and for municipalities with 25,000 inhabitants or more. Because of the disruptions of war and a delayed post-war recovery, no such bulletins were published at national level for the period July 1944 to December 1945. This reporting gap was filled by generating and combining additional information from local sources as identified next.

We added monthly mortality data for this period for the cities of Amsterdam, Rotterdam, and The Hague, obtained from the vital statistics reports published by these municipalities (Bureau van Statistiek der Gemeente Amsterdam 1945 and 1949; Gemeentelijk Bureau voor de Statistiek Rotterdam 1946; Bureau voor Statistiek en Voorlichting der Gemeente ‘s–Gravenhage 1946) and additional municipal estimates by Ekamper et al. (2020) and De Zwarte (2020).

To these sources we added national monthly mortality data by age for this period as published by CBS (1957). This last source also includes data on cause of death by age group but not by month or region/municipality. In 1945, there was a strong increase in reported deaths from unknown causes (‘ill-defined conditions’) compared with previous and later years. Overall, the share of unknown causes in all causes of death increased from 10 to 15 per cent in 1945 but in the 1–14 age group only from around 5 to 7 per cent. No increase was seen for infant deaths.

Due to the circumstances of war, information on the age-specific structure of the population in the years 1944–46 is missing for many municipalities in the Netherlands (CBS 1948). We therefore estimated the population structure by age and region for these years from the population of the Netherlands on 1 January in the years 1944–47 by age (year of birth) (CBS 1970); from population counts by region in the years 1944–47, available from the Historical Database of Dutch Municipalities (Boonstra 2016); and from population counts by year of birth and region, as available from the national 1947 Population Census. All three sources are based on CBS vital population statistics data and consistent with the CBS data framework. The first step in the estimation procedure was a backward ‘projection’ of the single-age cohorts from the 1947 Population Census to previous years by adding age-specific deaths. In the next step, the resulting initial population estimates by age, sex, and region for previous years were made consistent with the national population totals by age and sex and with the regional population totals by sex for these years, using (iterative) proportional fitting. The monthly population at risk in each year was calculated by linear interpolation of the average monthly population (for each age group in each region) between 1 January and 31 December of that year. Because not all data were available by sex, we did not differentiate between sexes in our analyses.

Although CBS tried to reconstruct vital population statistics for the later war years, the number of reported deaths and stillbirths might still be too low. Therefore, mortality rates, especially in the regions and time periods strongly affected by the famine or the liberation battle might be underestimated. The data may thus have some limitations, but current and past CBS publications on mortality and causes of death during the wartime period are all based on the reconstructed data and have been used previously for studies of famine and mortality (Ekamper et al. 2017). Any inaccuracies in the estimated populations at risk will affect mortality rates between regions in opposite directions: since the estimations are consistent within the national totals, underestimation of population at risk in one region implies overestimation in the other, resulting in overestimation of mortality rates in the one region and underestimation in the other. However, the impact on overall mortality patterns will be limited, since the populations at risk are relatively large compared with the number of deaths.

Our data set allowed us to extend the available CBS data by reconstructing monthly numbers of deaths and stillbirths by region over the period July 1944 to December 1945 and by estimating the monthly populations at risk by age group and region.

### Methods of analysis

We first evaluated age- and region-specific mortality in the years 1935–47, analysing infant and child mortality in the three famine-affected cities in the western Netherlands—Amsterdam, Rotterdam, and The Hague—relative to the rest of the Netherlands. We compared annual and monthly changes in overall death rates in age- and region-specific subgroups and calculated the number of deaths per 10,000 (average) population at risk in each age group. In this paper we follow the common usage in vital statistics to refer to this ratio as a rate, although rates express event densities in a defined time period (Elandt-Johnson 1975). We converted monthly rates to annual rates by multiplying monthly numbers by the number of days of the year divided by the number of days of that month. Age at death was categorized into the four groups (stillbirths, <1 year, 1–4 years, and 5–14 years) to compare the impacts of war and famine on different age groups.

Stillbirth rates were calculated as the number of stillbirths per 10,000 births (live plus stillbirths). Annual infant mortality rates (for ages <1 year) are usually calculated using the annual number of live births as the denominator. However, since we used monthly rates for infants aged <1 year, the number of monthly births was unsuitable as a denominator because it refers to children aged <1 month, making the age group of the numerator and denominator inconsistent. We therefore used the population aged <1 year as the population at risk. For mortality in the other age groups, we used the populations aged 1–4 and 5–14, respectively, as the denominators.

We then defined four time periods to examine infant and child mortality in the Hunger Winter in relation to secular changes between 1935 and 1947. We selected: (1) the pre-war period from January 1935 to April 1940; (2) the pre-famine period of wartime occupation from May 1940 to October 1944; (3) the famine period of occupation from November 1944 to May 1945; and (4) the post-war period after liberation, from June 1945 to December 1947. Mortality in each period was compared with the pre-war period as the reference. For each period we report in this paper the number of deaths by age group, separately for the three cities in the west and for the remainder of the Netherlands, and we express this number as deaths per 10,000 population at risk. We compared mortality in the three cities with mortality in the remainder of the Netherlands in all subgroups to evaluate whether there was an ‘urban advantage’, as previously reported by Hart (1993) for stillbirths.

Finally, we assessed age-specific causes of death in the years 1935–47. Cause of death was recorded only at the national level. As before, mortality rates are given separately here for deaths in the first year of life (infant mortality), deaths at ages 1–4, and deaths at ages 5–14.

## Results

### Annual mortality rates

**Figure 2:**
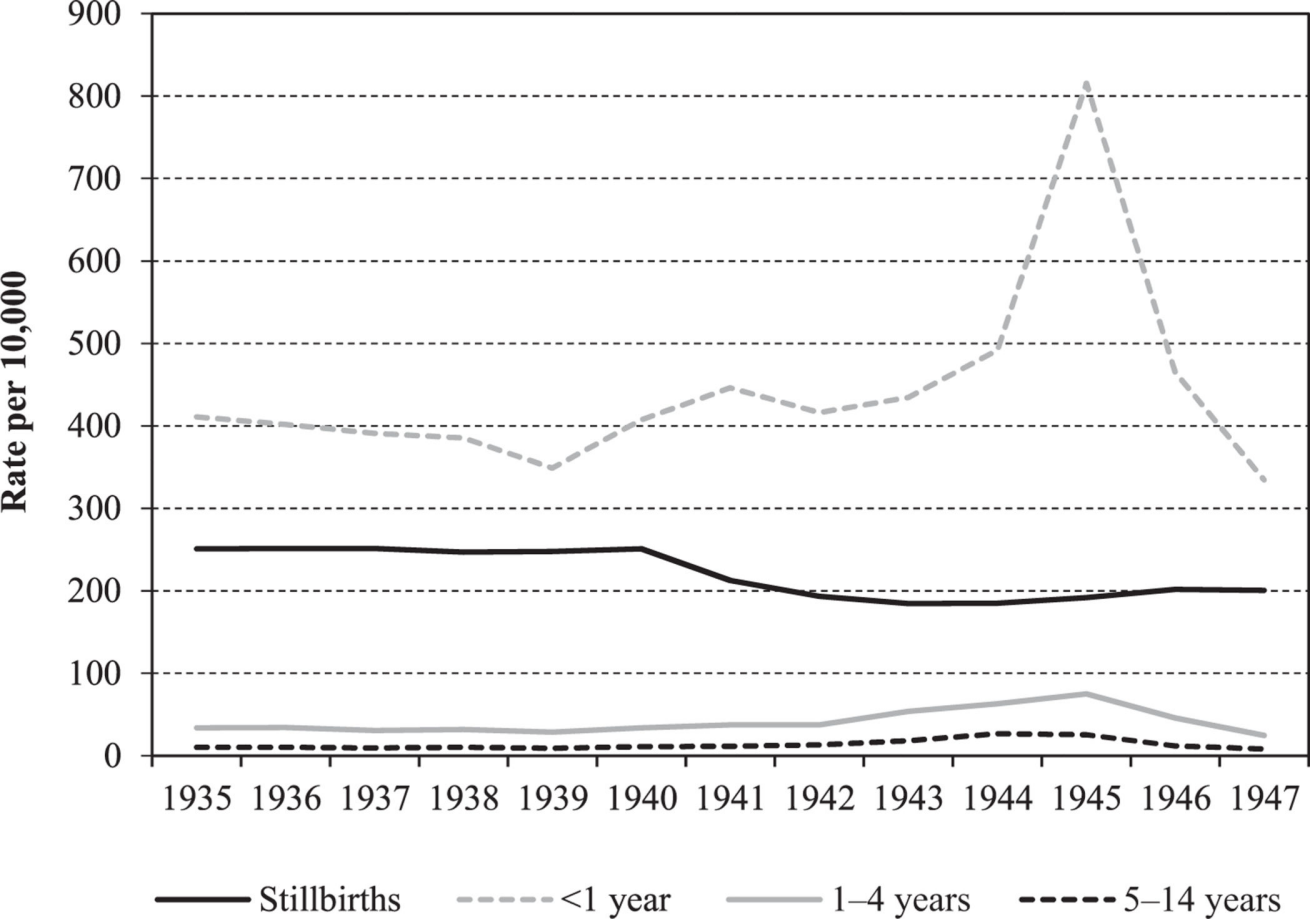
Annual mortality per 10,000 population at risk for selected age groups 0–14 and stillbirths, Netherlands, 1935–47 *Source*: CBS (1957).

The annual stillbirth rates and mortality rates for ages 0–14 (per 10,000 population at risk) for the period 1935–47 in the Netherlands are shown in Figure 2. To demonstrate the absolute mortality differential over time at different ages, mortality rates are shown separately for deaths in the first year of life (infant mortality), deaths at ages 1–4 (under-five mortality), and deaths at ages 5–14. Figure A1 in the supplementary material shows the relative changes in annual mortality per 10,000 population at risk over time at different ages on a log scale.

Figure 2 shows that in the pre-war years (1935–39), average mortality rates were highest in the first year of life (infant deaths 390/10,000) and for stillbirths (250/10,000) but were an order of magnitude lower for deaths at ages 1–4 and 5–14 (32/10,000 and 10/10,000, respectively).

Reported stillbirths show a gradual decline over time and were insensitive to the famine period. Infant deaths started to increase at the beginning of the war and doubled during the famine period. The relative impact of famine on mortality among children in the two older age groups appears to be small on this scale but is still noticeable. Mortality rates in these age groups in 1945 were 2.5 times higher compared with the pre-war period.

### Monthly mortality rates

**Figure 3:**
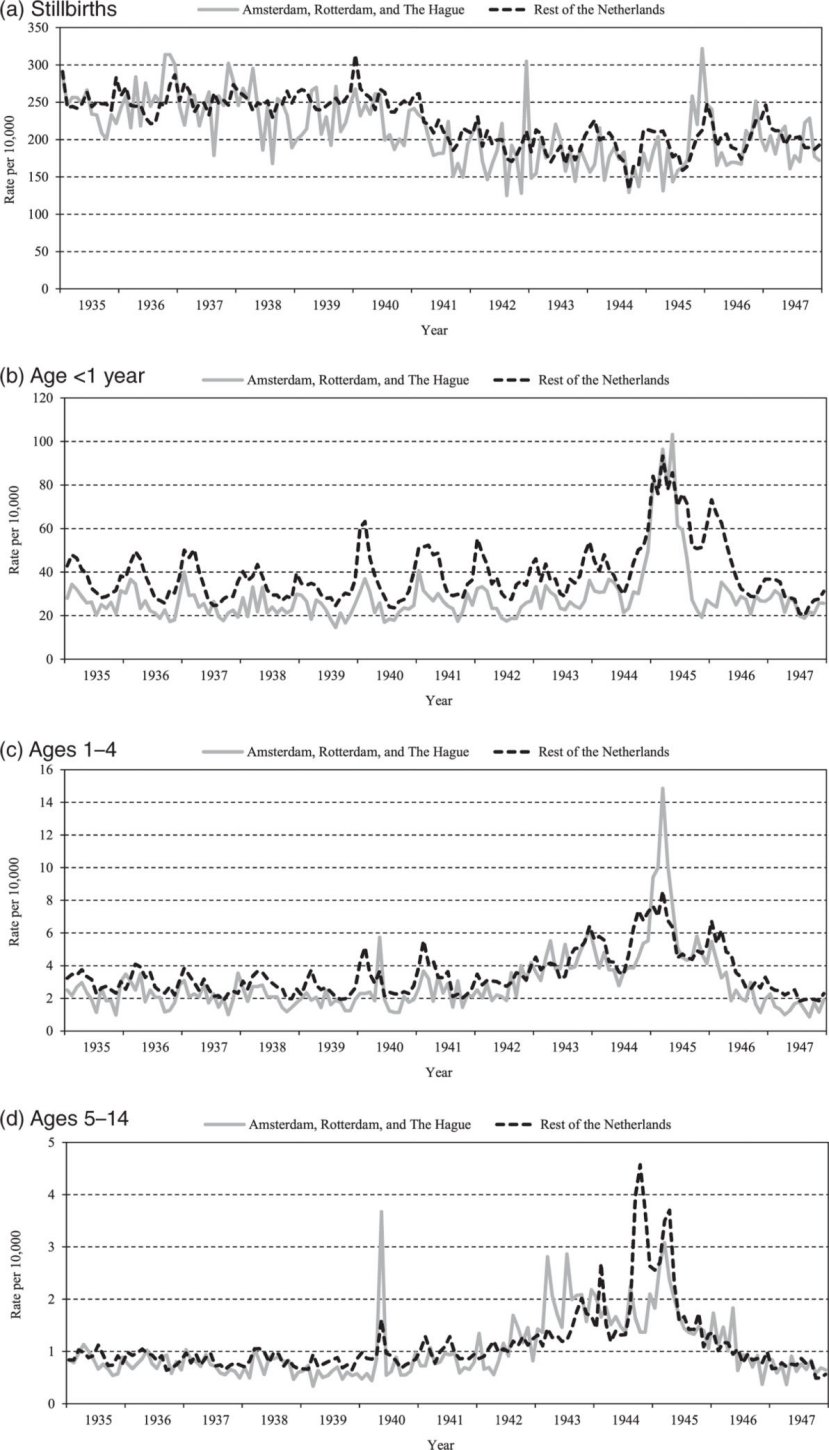
Monthly mortality per 10,000 population at risk for selected age groups 0–14 and stillbirths, three cities vs rest of the Netherlands, 1935–47 *Source*: Data compiled from CBS (1935–48), Bureau van Statistiek der Gemeente Amsterdam (1945, 1949), Bureau voor Statistiek en Voorlichting der Gemeente ‘s–Gravenhage (1946), Gemeentelijk Bureau voor de Statistiek Rotterdam (1946), Ekamper et al. (2020), and De Zwarte (2020).

More detailed mortality patterns are presented in Figure 3. For the period 1935–47, we show monthly stillbirth rates and monthly mortality rates for infants, children aged 1–4, and children aged 5–14, contrasting the rates in the three cities most affected by the famine (Amsterdam, Rotterdam, and The Hague) with rates in the remainder of the Netherlands.

Before the famine, mortality in all age groups tended to be lower in the three largest cities compared with the rest of the Netherlands, indicating an urban advantage. In absolute terms, the pre-famine urban advantage was largest for infant deaths (Figure 3(b)). Infant deaths in particular also show a strong seasonal pattern in both groups.

Relative peaks in stillbirth rates were seen in the three cities at the end of 1942 and the end of 1945 (Figure 3(a)). For infant deaths, peaks are observed in early 1945 (during the famine) in the three cities and in early 1945 and early 1946 (after the famine) in the remainder of the Netherlands (Figure 3(b)). Deaths at ages 1–4 peaked at the beginning of 1945, especially in the three cities (Figure 3(c)). Deaths at ages 5–14 peaked in May 1940 and 1943 in the three cities, at the end of 1944 in the remainder of the country, and in early 1945 in both areas (Figure 3(d)).

Compared with older children, infants aged <1 year experienced the highest mortality rates during the Hunger Winter, in both the three famine cities and the rest of the Netherlands (Figure 3(b)). At the height of the famine, in March 1945, infant mortality in the three famine-affected cities reached 105/10,000, which was about seven times higher than in the 1–4 age group (15/10,000) and over 30 times higher than in the 5–14 age group (3/10,000).

The proportional mortality increase during the famine period was highest in the 1–4 age group, with deaths in the famine cities in March 1945 seven times higher than usual compared with twice as high in the rest of the country (Figure 3(c)). In March 1945, infant mortality in the three famine cities was almost four times higher than usual and over twice as high as in the rest of the Netherlands (Figure 3(b)). In the 5–14 age group, mortality in the famine cities in early 1945 was three times higher than usual and 3.5 times higher than in the rest of the Netherlands (Figure 3(d)).

Although our data set did not allow for differentiation by sex, the study by Ekamper et al. (2017, pp. 120–4) provided some information on monthly mortality by sex over the years 1944–47 for the age groups <1 and 1–14 years. In general, mortality was 25–30 per cent higher in boys compared with girls. These differences were constant over time in the famine and non-famine regions. National statistics show a similar excess of deaths among males from the 1920s to the mid-1950s (CBS 1957). We assume that this sex-specific difference in child mortality persisted during the Hunger Winter.

### Mortality in specific time periods

Table 1 shows the reported number of deaths, mortality per 10,000 population at risk, absolute and relative changes in deaths relative to the pre-war period, and the urban advantage in absolute and relative terms in the four time periods (pre-war, pre-famine, famine, post-war). For the risk ratio (RR), we include a 95 per cent confidence interval (CI). As indicated by the narrow CIs around the RR estimates, the *p*-values are small for each estimate (*p* < 0.0001). Figure A2(a)–(d) in the supplementary material visualizes the most important findings from Table 1.

In all time periods except the famine period, we observe lower mortality in the three cities compared with the rest of the Netherlands (urban advantage). In the pre-war reference period, the largest urban advantage in absolute terms was for infant deaths, with a difference of 126/10,000 deaths (301/10,000 in cities vs 427/10,000 in the rest of the Netherlands). The absolute differences in the other age groups were much smaller, ranging from 1.5/10,000 deaths (ages 5–14) to 9/10,000 (ages 1–4) and 10/10,000 (stillbirths).

During the war, the urban advantage was maintained for stillbirths, which continued to show a declining trend throughout the occupation years in both the cities and the rest of the Netherlands, including during the famine period (November 1944 to May 1945). In the Hunger Winter, stillbirth rates were 30 per cent lower than in the pre-war period in the three cities (169/10,000 vs 243/10,000) against 20 per cent lower in the rest of the Netherlands (204/10,000 vs 253/10,000), therefore enlarging the urban advantage. However, in the immediate post-war period 1945–47, stillbirth rates rose in the three cities (to 192/10,000 vs 169/10,000 during the famine) while continuing to decline slightly in the rest of the Netherlands.

Infant death rates during the famine period increased threefold in the cities compared with pre-war rates (922/10,000 vs 301/10,000) and more than twofold in the rest of the Netherlands (908/10,000 vs 427/10,000). As Figure A2(c)–(d) (supplementary material) confirms, this shows that the urban advantage for infant deaths was lost during the famine. The urban advantage was also lost for the 1–4 age group, in which death rates increased over fourfold in the cities during the famine period (109/10,000 vs 26/10,000) but by only 2.5 times in the rest of the Netherlands (87/10,000 vs 35/10,000).

By contrast, the 5–14 age group maintained their urban advantage during the Hunger Winter. Compared with pre-war patterns, famine-period deaths among children aged 5–14 increased by a factor of three in the cities (27/10,000 vs 9/10,000) against 3.5 in the rest of the Netherlands (36/10,000 vs 10/10,000). Paradoxically, the urban advantage in this age group was therefore somewhat larger during the Hunger Winter than before or after the famine.

This urban advantage in the 5–14 age group is even maintained if we take into account the approximately 40,000 children from the urban western Netherlands who were evacuated between mid-January and mid-March 1945, of which an estimated 27,750 (70 per cent) were from Amsterdam and The Hague plus a small number from Rotterdam (De Zwarte 2016). If this number is subtracted from the three cities’ population at risk during the Hunger Winter (*n* = 152,411) and added to the rest of the Netherlands (*n* = 773,568), the currently reported urban advantage changes from a RR of 0.73 to a RR of 0.91, making the urban advantage less extreme and more in line with the urban advantage before and after the famine period. However, in reality, this shift in population at risk is probably too large, since the children were not evacuated throughout the Hunger Winter period and the age group of the evacuees (4–15) does not quite correspond with the age groups in our analyses (1–4; 5–14).

### Mortality by cause of death

**Figure 4:**
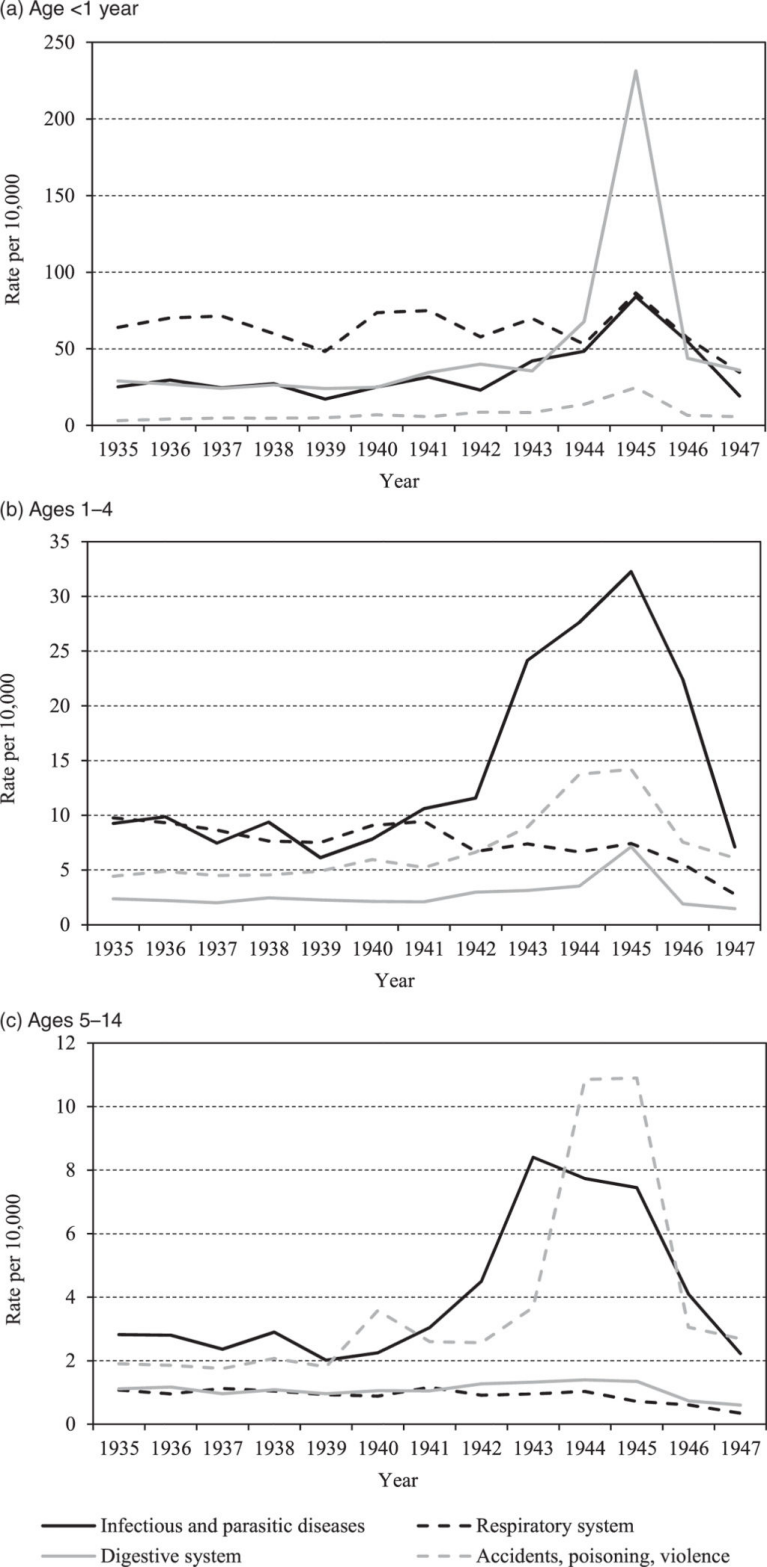
Annual mortality per 10,000 population at risk for selected age groups 0–14, by selected causes of death, Netherlands, 1935 *Source*: As for Figure 2.

Figure 4 shows annual infant and child mortality (per 10,000 population at risk) by cause of death in the Netherlands for the period 1935–47. It gives the four most important causes of death from the original 15-category classification (CBS 1957). The selected causes are infectious and parasitic diseases (black continuous line); digestive system diseases (grey continuous line); respiratory system diseases (black dotted line); and deaths from accidents, poisoning, and violence (grey dotted line).

For the <1 year age group (infant deaths; Figure 4 (a)), the increase in mortality from diseases of the digestive system during the famine is striking. In 1945, reported death rates from digestive system diseases were about nine times higher than in 1935–40. Mortality by other causes was also higher during the famine but less strikingly so.

Among the much lower death rates in the 1–4 age group (Figure 4(b)), the most striking change during the famine period is the fourfold increase in death rates from infectious and parasitic diseases, rising to over 30/10,000. In addition, death rates from accidents, poisoning, and violence and death rates from diseases of the digestive system increased threefold.

Considering the even lower death rates in the 5–14 age group (Figure 4(c)), death rates from accidents, poisoning, and violence rose over fivefold during the famine period compared with the pre-war years, to 11/10,000. During the war, a threefold increase can be seen in death rates from infectious and parasitic diseases, rising to 8/10,000 in 1943 and then declining to about 7/10,000 during the famine year, 1945. There was no strong increase in death rates from diseases of the digestive system at ages 5–14 as seen at younger ages.

Deaths from ‘hunger or thirst’ were reported as a separate category in the classification system. Death certificates in the 1944–45 period listed a total of 8,290 famine deaths across all ages (see Table A1, supplementary material). The first deaths from starvation were reported in November 1944. The number rose sharply after January 1945 and reached a maximum in March 1945. The last hunger deaths were reported in July 1945, almost two months after liberation. Only 0.5 per cent of the hunger deaths were reported to be among children aged 5–14 (40 deaths), 0.9 per cent for children aged 1–4 (75 deaths), and 1.6 per cent for infants <1 year (135 deaths). Even though malnutrition-related mortality was most likely about three times higher than officially reported, the number of reported famine deaths was relatively small (Ekamper et al. 2017).

## Discussion

Using information from vital statistics reports in the Netherlands for the period 1935–47, we have provided a detailed description of the impact of the Dutch Hunger Winter famine on infant and child mortality based on national data. The data may have some limitations due to less complete registration of population and deaths at that time, particularly during the last stages of the war. However, the registration system remained largely functional. Incomplete registration of deaths will, moreover, lead to estimated losses that are biased downwards. This makes our estimates of wartime losses conservative. No longer restricted to using only aggregated, annualized deaths and shifts in proportional mortality from specific causes, we reported age-specific overall mortality and causes of death among well-defined populations at risk in different age groups. For each age at death, we compared mortality during the Dutch Hunger Winter with mortality before and after the famine, separately for the three largest famine cities and for the remainder of the country. In this section we discuss three important elements of the famine’s impact: (1) the absolute and relative vulnerability of different age groups to famine; (2) trends in causes of death; and (3) the urban advantage in mortality rates.

### Absolute and relative vulnerability to famine

We first examined which age groups were most vulnerable to famine in absolute and relative terms. In most historical famines, the highest death rates in absolute terms have been reported in infants and young children (ages 0–4) and in older people, whereas the largest proportional mortality increase is often reported for later childhood ages. The age-specific mortality patterns during the Dutch famine are not fully in line with these previous findings.

In absolute terms, our data confirm that infants experienced the highest mortality during the famine. In the cities, infant mortality increased to 922/10,000 compared with 109/10,000 for deaths at ages 1–4 and 27/10,000 for deaths at ages 5–14. Over 60 per cent of deaths between ages 0 and 14 took place in the first year of life.

In relative terms, however, all three age groups experienced a three/fourfold increase in mortality during the famine period. This is not in line with previous observations that older children experience the highest proportional mortality during famines. During the Finnish famine of the 1860s and the Ukrainian famine of 1932–33, the proportional mortality increases among children aged 5–14 years were three to nine times *higher* than they were for infants (e.g. Pitkänen and Mielke 1993; Vallin et al. 2002). During the Hunger Winter all age groups experienced the *same* three/fourfold relative increase.

Regarding stillbirths, we saw a constant decline in mortality rates over time, both in the three cities and in the rest of the Netherlands. There was, however, a transient additional decline in stillbirths during the famine in the large western cities with no clear explanation. This additional decline was based on small numbers and could have resulted from random variation, falling stillbirth registration during the famine period, or both.

### Changes in causes of death

We further examined the famine’s impact by looking at causes of death in specific age groups. Previous studies have shown that a large part of the mortality increase during Second World War European famines, including the Hunger Winter, was due to starvation and malnutrition-related diseases including tuberculosis, diarrhoea, and diseases of the digestive and circulatory systems (Hionidou 2002; Ó Gráda 2010). Excess deaths from infectious and digestive diseases in the Netherlands were not the result of social disruptions associated with famine (De Waal 1989), as there was neither mass migration nor increased mobility during the Hunger Winter. That the Netherlands was spared from major epidemics during the famine can largely be attributed to a healthcare system that already included effective measures for maintaining levels of hygiene and preventing the spread of infectious disease (De Zwarte 2020).

Our data showed that the impact of the famine was largest among infants (<1 year), where diseases of the digestive system showed a tenfold mortality increase. Most deaths in this category were from enteritis, diarrhoea, and ulceration of the intestines, all of which are important signals of famine (Dirks 1993; Hionidou 2002). Deaths from digestive diseases also increased in the 1–4 age group, where a threefold increase was seen.

In the 1–4 and especially in the 5–14 age groups, non-famine-related war activities made up a significant proportion of the excess deaths. Specific mortality peaks at these ages (Figure 3(c)–(d)) can be explained by the German and Allied bombings in Rotterdam (May 1940, May 1943), Amsterdam (July 1943), and The Hague (March 1945) that killed many civilians (Hogervorst and van Ulzen 2015). In the remainder of the Netherlands, the mortality peaks in October 1944 and in April 1945 correspond to combat activities during the liberation of the south of the Netherlands and of the north-eastern provinces, both accompanied by heavy fighting and loss of life (Ekamper et al. 2017).

The spike in death rates from infectious diseases in the 1–4 and 5–14 age groups during 1942–45 (Figure 4(b)–(c)) can be attributed largely to diphtheria mortality, although numbers of deaths remained relatively low in absolute terms. Futselaar (2008) connected this increase in diphtheria deaths to deteriorating wartime diets lacking vital micronutrients for health and growth. However, this explanation is not consistent with our observation that deaths from infectious diseases at ages 5–14 *declined* during the famine period. More likely, these mostly diphtheria deaths were connected to a European-wide epidemic that had already reached its peak before the Hunger Winter, combined with the cessation of mass immunization during the occupation (Van Wijhe et al. 2016). In 1945, about two-thirds of Dutch children aged six months to 12 years had not been immunized against diphtheria (Stuart 1945).

The relative vulnerability to famine was therefore closely connected to age-specific causes of death. The most common primary causes of death among older children (i.e. accidents, poisoning, and violence) were not directly connected to starvation or its related diseases. This explains why the impact of famine on mortality among older children was limited. By contrast, the famine had a specific impact on infant mortality from diarrhoea and enteritis through the combined effects of decreasing birthweights among infants exposed to famine in late gestation and the postnatal exposure to summer epidemics affecting mostly infants and young children (Stein et al. 1975). For these reasons, the mortality effects of undernutrition during the Hunger Winter were confined largely to the younger age groups.

Neurdenburg (1947, p. 388) stated that the large increase in infant deaths from digestive system diseases was due to lack of hygiene, in particular ‘ineffective nutrition and difficulties with preparing meals’ (see also Trienekens 1985). The lower ability of malnourished women to breastfeed—suggested by reduced post-partum maternal weight (−4.3 per cent) and reduced placental weight (−15 per cent) (Stein and Susser 1975), leading to earlier weaning and exposure to contaminated foods—may also have contributed to these trends. However, the lowered resistance of infants with lower birthweights (by about 300 g or −9 per cent) because of the famine most likely also contributed to increased mortality from these conditions (Smith 1947; Sindram 1953; Stein et al. 1975; Futselaar 2008). German policies did not play a role in the reported trends in mortality, as the occupation authorities did not interfere with rationing nor healthcare measures during the famine period (De Zwarte 2020).

The trends in reported stillbirths over time in the famine-affected cities and in the rest of the Netherlands (Figure 2) are consistent with Hart’s (1993) observation that stillbirths were relatively little affected by the Hunger Winter. Higher rates of miscarriage during the famine period may have had an impact on stillbirths, but this relationship is impossible to determine due to lack of data. Birth outcomes appear to show little relationship to the significant fertility changes related to the famine or to the sex ratio, which was not affected (Cramer and Lumey 2010). As analysed by Stein et al. (1975), the number of births conceived under famine conditions (and hence born after the war) was one-third lower than would have been expected under normal conditions. At age 18, the number of military recruits was 50 per cent lower (Ekamper et al. 2014). While infant death rates during the famine period were significantly higher than in non-famine periods, the increase was driven entirely by deaths in the first three months of life and was not seen for mortality at ages 90–365 days (Stein et al. 1975). The increase in deaths to infants born during the famine therefore relates to conceptions before the famine period. Births conceived under famine conditions and born after the war show no increase in infant or later mortality. We therefore see no selection effect on birth outcomes in relation to changes in fertility. Reliable data are lacking to examine if the decline in births observed by Stein et al. (1975) could have resulted from reductions in conceptions and/or increases in miscarriages.

For older children, our data showed that the impact of the famine was smaller than previously assumed. If we removed deaths from accidents, poisoning, and violence from the mortality count, the proportional mortality increase in the 5–14 age group would even have been *lower* than among infants. This is contrary to findings from most other European famines. A possible explanation for our finding is the effectiveness of relief interventions in the Netherlands (i.e. emergency nutrition, evacuation). These non-governmental activities were allowed by the German occupier and specifically targeted the most malnourished school-aged children, including half of the child population at risk (De Zwarte 2018, 2020). Our findings are in line with earlier reports on the possible relationship between child mortality patterns and relief interventions during the Ukrainian and Greek famines (Davies and Wheatcroft 2004; Hionidou 2006). Dutch relief efforts began to include young children (aged 1–4) two months later than schoolchildren. This possibly relates to our finding that deaths in the 1–4 age group showed clear signs of links to famine in addition to other war-related causes of death. For infants and pregnant women, rations and relief proved insufficient and were unable to prevent lower birthweights and lowered resistance (Stein et al. 1975), thereby increasing infants’ susceptibility to infectious and digestive system diseases.

### The urban advantage

We confirmed lower urban stillbirth rates (urban advantage) as described by Hart (1993) in the famine region in the years 1944–46. While Hart’s data included only stillbirths in this limited time window, we also examined infant deaths and childhood deaths at ages 1–4 and 5–14 in the period 1935–47 and compared mortality in the three largest famine cities with mortality in the remainder of the country.

The urban advantage for stillbirths was not limited to 1944–46 but could be seen over the entire period 1935–47. The urban advantage was also seen for infant deaths and for mortality at ages 1–4 and 5–14 in all years except during the famine period. We speculate that the urban advantage arose from a combination of better access to healthcare and vaccination programmes, improved hygiene and sanitation practices, and better education among the city populations. These factors had caused mortality rates in the Netherlands to steadily decline since the 1870s, especially mortality from infectious diseases (Van Poppel et al. 2005).

The urban advantage was counteracted by the famine exposure of the city population in the period November 1944 to May 1945. In this period, infant deaths and mortality among children aged 1–4 was higher in the three cities compared with the rest of the Netherlands, in both absolute and relative terms. Paradoxically, the urban advantage was not lost but even *increased* during the Hunger Winter for deaths at ages 5–14, even when taking into account population movements because of the child evacuation programmes. This suggests that excess deaths in this age group during the famine were not necessarily connected to starvation or related diseases.

## Conclusion

Our study has illustrated the need to examine absolute and relative mortality changes combined with age-specific causes of death for a comprehensive understanding of the impact of a famine. The number of famine deaths will be relevant for quantifying a famine’s impact at the population level and determining the required scale of intervention. Increases in relative mortality will help to identify population subgroups at risk and target specific interventions. Ideally, both mortality measures will relate to a well-defined population at risk.

In the Dutch Hunger Winter, the impact of the famine was most visible in infants <1 year of age. This group showed the highest mortality rate, exceeding 900/10,000, a high proportional mortality (threefold increase during the famine), and increased mortality specifically from digestive system diseases related to famine exposure. This makes infant mortality a desirable indicator of famine severity in similar acute settings and a useful marker for comparative use in future studies on famine mortality.

In the 1–4 and especially in the 5–14 age groups, mortality trends were less directly connected to the famine and more related to the violence of war, which made up a significant proportion of the excess deaths. Our findings also showed that during the famine the increased mortality risk among children aged 5–14 was lower in the famine-affected cities than elsewhere in the Netherlands, thereby increasing the pre-famine urban advantage. Therefore, mortality at ages 1–4 and 5–14 are not suitable indicators of famine severity in this setting.

In summary, infant mortality in our setting was the most sensitive indicator of famine severity, with the largest number of deaths and the most direct link between famine exposure and famine-specific causes of death. We expect that our findings will apply to other nineteenth- and twentieth-century European famines as well, but this would need further confirmation. Further investigations of infant and child mortality in different settings will be important for a better understanding of vulnerability and resilience to famine today and in future and also for shaping adequate relief interventions.

## Supplementary Material

Supplementary Material

## Figures and Tables

**Table 1:** Number of deaths, population at risk, and mortality per 10,000 population at risk at selected ages 0–14 years and stillbirths, in three cities vs rest of the Netherlands, for four time periods, Netherlands, 1935–47

	Amsterdam, Rotterdam, and The Hague					Rest of Netherlands					Cities vs Rest	
Age group Period	Deaths	Population at risk	Deaths/10,000 population at risk	RR	95 per cent CI	Deaths	Population at risk	Deaths/10,000 population at risk	RR	95 per cent CI	RR	95 per cent CI
*Stillbirths* Pre-war: 1935 to Apr 1940	3,942	162,061	243.2	1.00	–	19,747	779,505	253.3	1.00	–	0.96	0.93–0.99
Pre-famine: May 1940 to Oct 1944	2,841	151,855	187.1	0.77	0.73–0.81	15,030	735,896	204.2	0.81	0.79–0.82	0.92	0.88–0.95
Famine: Nov 1944 to May 1945	393	23,253	169.0	0.69	0.63–0.75	2,285	112,182	203.7	0.80	0.77–0.84	0.83	0.75–0.92
Post-war: Jun 1945 to 1947	2,405	125,480	191.7	0.79	0.75–0.83	10,756	536,004	200.7	0.79	0.77–0.81	0.96	0.91–1.00
*Infant deaths (<1 year)* Pre-war: 1935 to Apr 1940	4,691	155,956	300.8	1.00	–	30,704	718,617	427.3	1.00	–	0.70	0.68–0.73
Pre-famine: May 1940 to Oct 1944	4,612	144,997	318.1	1.06	1.02–1.10	30,403	669,273	454.3	1.06	1.05–1.08	0.70	0.68–0.72
Famine: Nov 1944 to May 1945	1,849	20,046	922.4	3.07	2.91–3.24	8,772	96,636	907.7	2.12	2.07–2.18	1.02	0.97–1.07
Post-war: Jun 1945 to 1947	4,285	126,092	339.8	1.13	1.08–1.18	22,913	477,475	479.9	1.12	1.10–1.14	0.71	0.69–0.73
*Deaths 1*–*4 years* Pre-war: 1935 to Apr 1940	1,516	594,864	25.5	1.00	–	9,812	2,842,666	34.5	1.00	–	0.74	0.70–0.78
Pre-famine: May 1940 to Oct 1944	2,005	521,722	38.4	1.51	1.41–1.61	11,264	2,495,878	45.1	1.31	1.27–1.34	0.85	0.81–0.89
Famine: Nov 1944 to May 1945	746	68,595	108.8	4.27	3.91–4.66	2,965	341,465	86.8	2.52	2.41–2.62	1.25	1.16–1.36
Post-war: Jun 1945 to 1947	1,100	359,653	30.6	1.20	1.11–1.30	6,676	1,608,725	41.5	1.20	1.17–1.24	0.74	0.69–0.79
*Deaths 5*–*14 years* Pre-war: 1935 to Apr 1940	1,413	1,634,813	8.6	1.00	–	7,163	7,079,792	10.1	1.00	–	0.85	0.81–0.90
Pre-famine: May 1940 to Oct 1944	1,976	1,262,016	15.7	1.81	1.69–1.94	9,424	5,965,465	15.8	1.56	1.51–1.61	0.99	0.94–1.04
Famine: Nov 1944 to May 1945	405	152,411	26.6	3.07	2.75–3.43	2,811	773,568	36.3	3.59	3.44–3.75	0.73	0.66–0.81
Post-war: Jun 1945 to 1947	817	698,284	11.7	1.35	1.24–1.48	4,054	3,438,963	11.8	1.17	1.12–1.21	0.99	0.92–1.07

*Notes:* RR = risk ratio; CI = confidence interval.

*Source:* Data compiled from CBS (1935–48), Bureau van Statistiek der Gemeente Amsterdam (1945, 1949), Bureau voor Statistiek en Voorlichting der Gemeente ‘s–Gravenhage (1946), Gemeentelijk Bureau voor de Statistiek Rotterdam (1946), Ekamper et al. (2020), and De Zwarte (2020).

## Data Availability

The data that support the findings of this study are available on request from the authors. The data are compiled from non-digitized public sources and also data that are not publicly available and subject to Statistics Netherlands restrictions. Restrictions apply to the redistribution and availability of these data, which were used in part under licence for this study.
