# Supplementary material for: Infant and child mortality in the Netherlands 1935–47 and changes related to the Dutch famine of 1944–45: A population-based analysis
Source: Popul Stud (Camb). Author manuscript; Available in PMC 2025 Nov 1. (PMC10927613; doi:10.1080/00324728.2023.2243913)
Supplement: Supplementary Material [file NIHMS1934406-supplement-Supplementary_Material.pdf]

Infant and child mortality in the Netherlands 1935–47 and changes related to the Dutch Hunger Winter of 1944-45: A population-based analysis

Annex

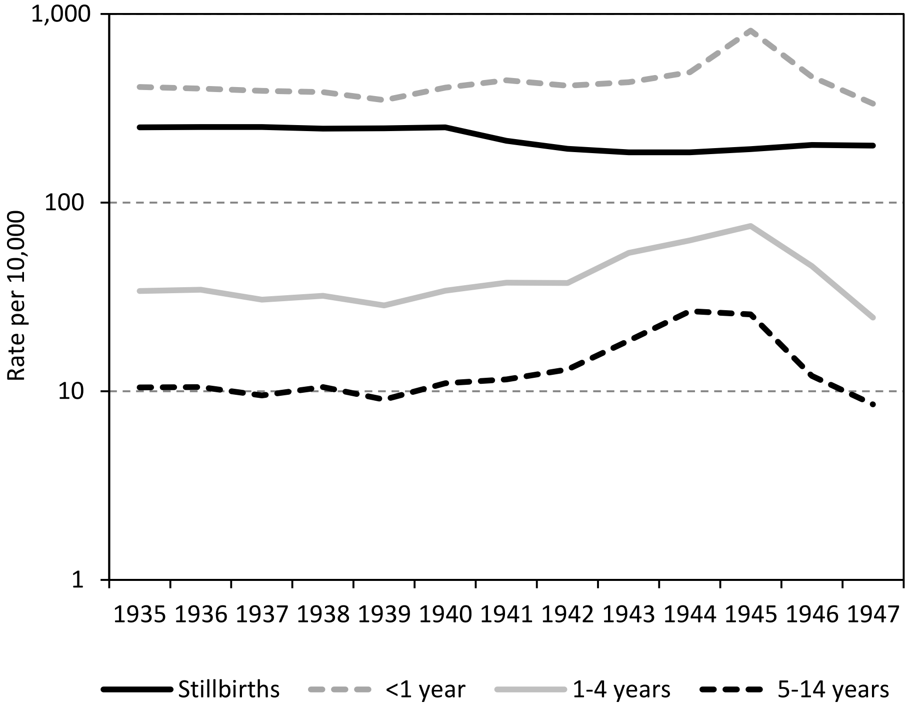

Figure A1. Annual mortality per 10,000 at risk (log scale) at selected ages 0-14 years and stillbirths, Netherlands, 1935–1947

Source: CBS (1957).

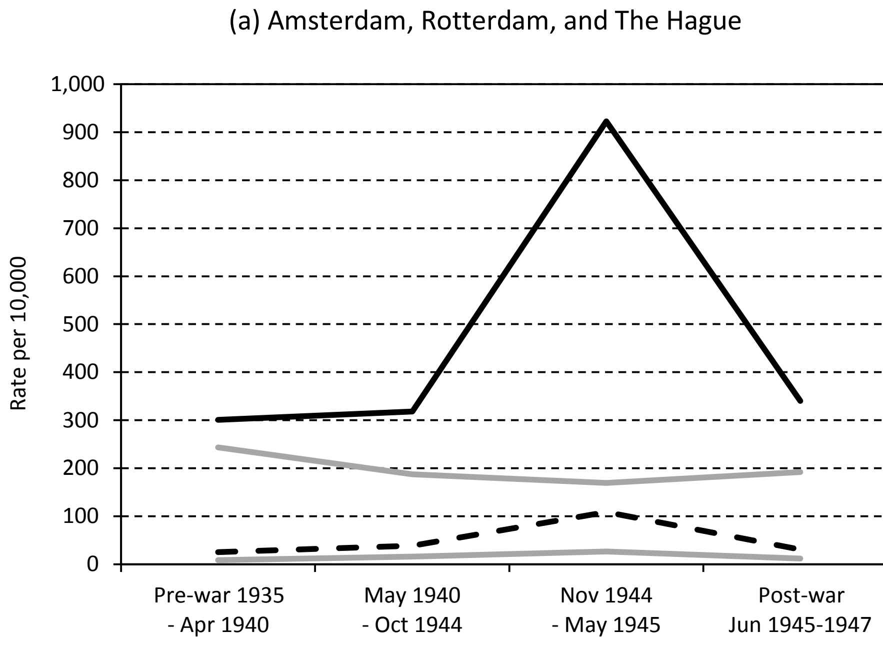

(a) Amsterdam, Rotterdam, and The Hague
Rate per 10,000
1,000
900
800
700
600
500
400
300
200
100
0
Pre-war 1935 - Apr 1940
May 1940 - Oct 1944
Nov 1944 - May 1945
Post-war Jun 1945-1947

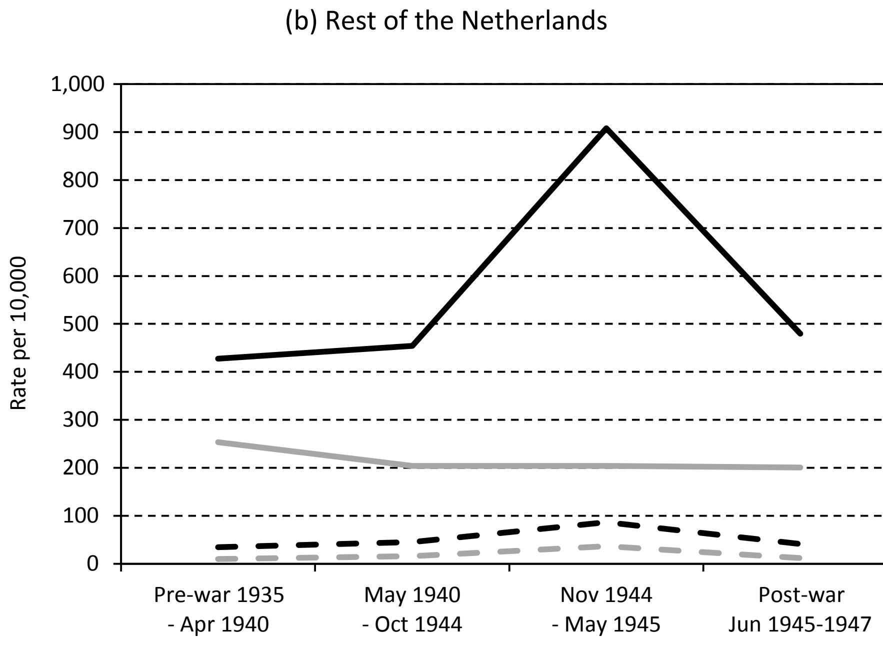

(b) Rest of the Netherlands
Rate per 10,000
1,000
900
800
700
600
500
400
300
200
100
0
Pre-war 1935 - Apr 1940
May 1940 - Oct 1944
Nov 1944 - May 1945
Post-war Jun 1945-1947

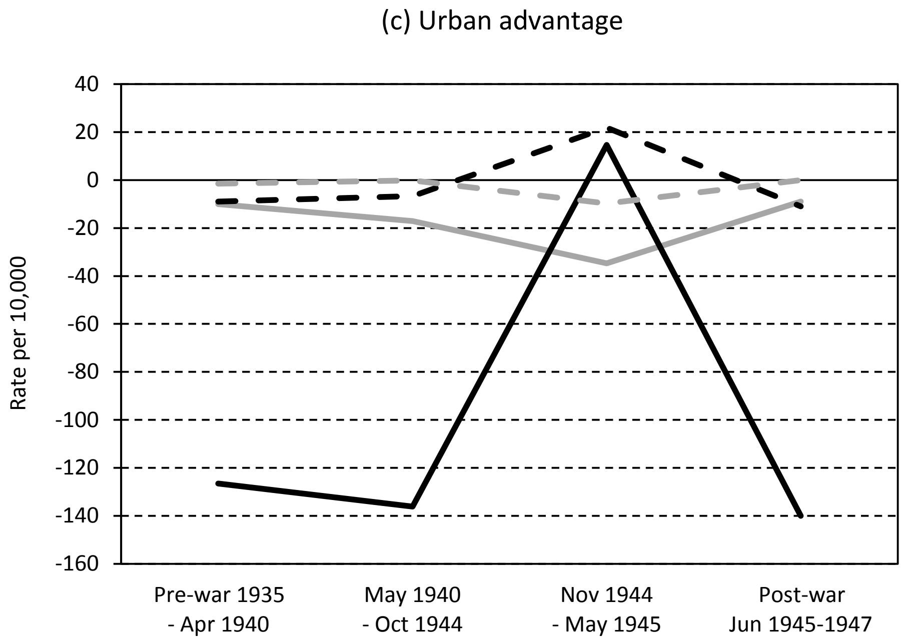

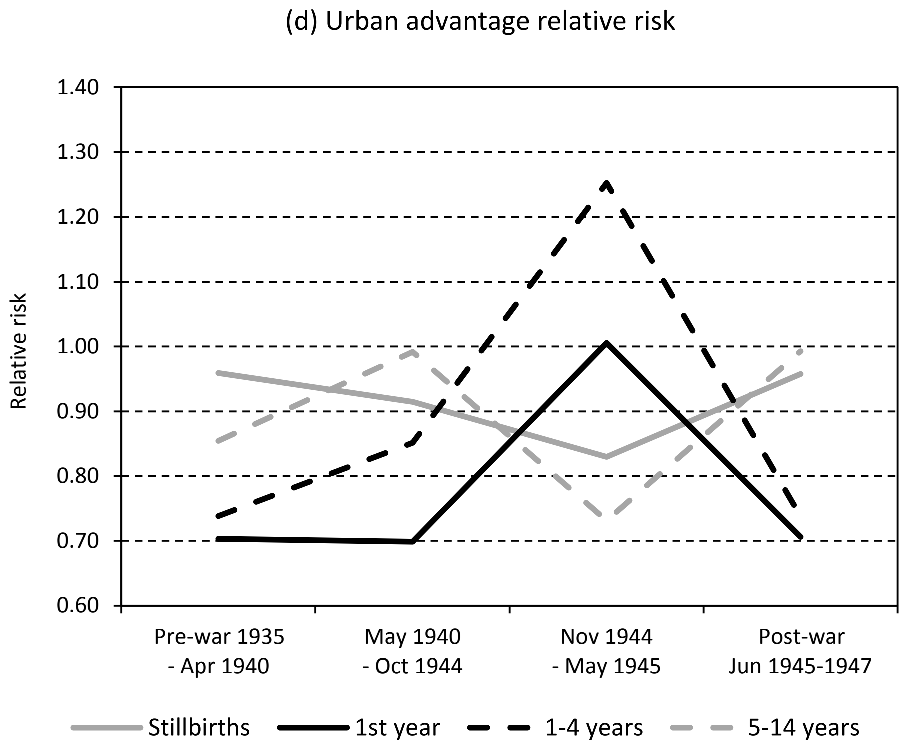

Figure A2. Mortality per 10,000, urban advantage, and urban advantage relative risk at selected ages 0-14 years and stillbirths by region for four time periods, Netherlands, 1935–1947

Source: data compiled from CBS (1935-48), Bureau van Statistiek der Gemeente Amsterdam (1945, 1949), Bureau voor Statistiek en Voorlichting der Gemeente ’s Gravenhage (1946), Gemeentelijk Bureau voor de Statistiek Rotterdam (1946), Ekamper et al. (2020), and De Zwarte (2020).

Table A1. Number of reported famine deaths by age group and region, Netherlands, 1944–45

	Deaths	%
Age groups		
0	135	1.6
1-4	75	0.9
5-9	25	0.3
10-14	15	0.2
15-19	25	0.3
20-24	45	0.5
25-54	1,450	17.5
55-69	3,150	38.0
70+	3,370	40.7
Regions		
Urban West	6,800	82.0
Rural West	1,320	15.9
Rest Netherlands	170	2.0
Total	8,290	100.0

Notes: Cause of death ICD5 code 189 (hunger or thirst); numbers rounded to the nearest 5; excluding age and region unknown (officially reported number of famine deaths in 1944ς45 is 8,305).

Source: Calculations by NIDI using non-public microdata from Statistics Netherlands.
